# Specific Medication

**Published:** 1902-07

**Authors:** 


### Specific Medication.

Andrew H. Smith, in the *Medical Record* of March 15th, says, in substance, that those diseases caused by the presence of bacilli in body are marked by the generation therein of toxins. If these toxins can be kept from forming, the danger from the disease will be nil. The action of our various specifics is vital and not chemical in nature, thus resembling the action of the blood serum itself as an inhibitor of germ growth. Much has really been done along this line of inhibiting cell growth in various specific diseases. Pneumonia, when treated by sodium salicylate or creasote carbonate, usually recovers by lysis within three or four days, a complete resolution taking place. Influenza is favorably influenced by large doses of carbolic acid, the various sequelæ being kept at a minimum by this treatment. The same drug administered to the stage of carboluria has proven to be of marked value in scarlet fever. This treatment is rarely ever followed by renal troubles. The success of specific medication in these diseases leads us to expect great advances along the same lines in the future. C. R. M.

